# Biopsy strategies in the era of mpMRI: a comprehensive review

**DOI:** 10.1038/s41391-024-00884-2

**Published:** 2024-09-04

**Authors:** Olivier Windisch, Massimo Valerio, Chi-Hang Yee, Paolo Gontero, Baris Bakir, Christof Kastner, Hashim U. Ahmed, Cosimo De Nunzio, Jean de la Rosette

**Affiliations:** 1 Division of Urology, Geneva University Hospitals, Geneva, Switzerland; 2 Faculty of Medicine, Geneva University, Geneva, Switzerland; 3 SH Ho Urology Centre, The Chinese University of Hong Kong, Pok Fu Lam, Hong Kong; 4 Division of Urology, Department of Surgical Sciences, San Giovanni Battista Hospital, University of Studies of Torino, Torino, Italy; 5 Department of Radiology, Istanbul University, Istanbul Medical School, Istanbul, Turkey; 6 Department of Urology, Cambridge University Hospitals and University of Cambridge, Cambridge, UK; 7 Imperial Prostate, Department of Surgery and Cancer, Faculty of Medicine, Imperial College London, London, UK; 8 Imperial Urology, Imperial College Healthcare NHS Trust, London, UK; 9 Department of Urology, University Sapienza, Rome, Italy; 10 Istanbul Medipol University, Istanbul, Türkiye; 11 Bashkir State Medical University, Ufa, Russia

**Keywords:** Cancer screening, Prostate cancer

## Abstract

**Background:**

Since its initial description the prostate biopsy technique for detection of prostate cancer (PCA) has constantly evolved. Multiparametric magnetic resonance imaging (mpMRI) has been proven to have a sensitivity exceeding 90% to detect the index lesion. This narrative review discusses the evidence around several biopsy strategies, especially in the context of patients that might be eligible for focal therapy.

**Method:**

A non-systematic literature research was performed on February 15th 2024 using the Medical Literature Analysis and Retrieval System Online (Medline), Web of Science and Google Scholar.

**Results:**

The transrectal (TR) route is associated with an increased postoperative sepsis rate, even with adequate antibiotic prophylaxis. The transperineal (TP) route is now recommended by international guidelines, firstly for its decreased rate of urosepsis. Recent evidence shows a non-inferiority of TP compared to TR route, and even a higher detection rate of clinically significant PCA (csPCA) in the anterior and apical region, that are usually difficult to target using the TR route. Several targeting techniques (cognitive, software-fusion or in-bore) enhance our ability to provide an accurate risk assessment of prostate cancer aggressiveness and burden, while reducing the number of cores and reducing the number of clinically insignificant prostate cancer (ciPCA). While MRI-TB have proven their role, the role of systematic biopsies (SB) is still important because it detects 5–16% of csPCA that would have been missed by MRI-TB alone. The strategies of SB depend mainly on the route used (TR vs. TP) and the number of cores to be collected (10–12 cores vs. saturation biopsies vs. trans-perineal template mapping-biopsies or Ginsburg Protocol vs. regional biopsies).

**Conclusion:**

Several biopsy strategies have been described and should be known when assessing patients for focal therapy. Because MRI systematically under evaluates the lesion size, systematic biopsies, and especially perilesional biopsies, can help to increase sensitivity at the cost of an increased number of cores.

## Introduction

**Fig. 1:**
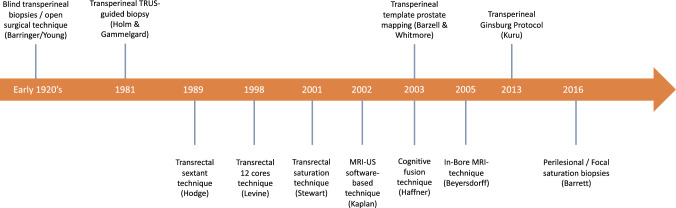
Timeline of biopsies. Figure 1 provides a timeline of the different biopsy techniques described in the procedure. Name in parenthesis correspond to the first author of the technique first description [1, 49, 51, 52, 77–82].

Since its first description in the early 1920s, by transperineal open surgery, prostate biopsy techniques have evolved fast towards less invasive, less morbid, and more accurate sampling. The first description of the transrectal approach using the sextant transrectal biopsy with ultrasound guidance was reported by Hodge in 1989 [1]. After this first description, the 10–12 cores became the standard method because of the acceptable balance of increased detection rate and acceptable side-effect rate compared to high core numbers [2]. The prostate biopsy evolution timeline is displayed in Fig. 1. A randomized-controlled trial (RCT) published in 1990 initially showed no benefit of prostate MRI over transrectal ultrasound, both modalities significantly underestimating prostate cancer risk, limiting at that moment its adoption. Since then, prostate multiparametric magnetic resonance imaging (mpMRI) gained significant attention after reports showing improved cancer detection. A 30-fold increase in the use of mpMRI before biopsy was observed in the US from 2009 to 2015 (from 0.2% to 6.5%) even before formal recommendation was formulated [3]. Since then, growing evidence supports the routine use of mpMRI; the PROMIS trial showed a sensitivity to detect clinically significant prostate cancer (csPCA) exceeding 90%, while the PRECISION trial showed the ability of mpMRI targeted biopsy (MRI-TB) to increase the detection of csPCA while decreasing the detection of clinically insignificant prostate cancer (ciPCA) and at the same time avoiding the need for biopsies in 28% of the patients [4, 5]. The European Association of Urology (EAU) recommends mpMRI as an upfront tool to guide biopsies in biopsy-naïve patients and for patients with previous negative systematic biopsies with persistent cancer suspicion [6].

The advance of mpMRI has changed the prostate cancer diagnostic pathway. Prior to MR-imaging, the debate around prostate biopsy concerned density and location. The 10–12 cores transrectal ultrasound guided biopsy became the pragmatic response to sample the area of the prostate more likely to harbor PCA, namely the peripheral zone, in a systematic fashion from the base to the apex. A prostate MRI prior to biopsy offers clinicians an imaging phenotype which prompts different questions to be addressed. First, after the identification of a MR-visible lesion, the need for a precise targeting is required to realize targeted biopsies (TB). The distribution and density of targeted biopsies in and around a lesion are still matters of debate, which are particularly important in the context of focal therapy. Second, the value of additional systematic biopsy as well as the intensity and the route of sampling are key questions to be answered.

This manuscript aims to provide a comprehensive review of the most commonly used biopsy strategies reported in literature. While some are now considered obsolete by many, they will be included to provide a wide overview and to better assess the comparative diagnostic performance against technological evolutions. The manuscript will first focus on targeted biopsies, perilesional biopsies and the several types of guidance that the MRI information offers (cognitive, TRUS-MRI fusion or MRI in-bore), then discuss the role of systematic biopsies and combined biopsies. Safety aspects will be then discussed. The performance of these strategies in adequately identifying patients for FT concludes the discussion. Pros and conns of each technique are summarized in Table 1.

**Table 1:** Pros and Cons summary of biopsy techniques discussed in the review.

	Number of cores taken	ciPCA detection	csPCA detection	Advantages / Disadvantages	Feasible under local anesthesia?	Safety profile
**Transrectal**				Easy, quick, cheap, less equipment / Increased risk of urosepsis, difficulties in targeting anterior, periurethral and apical tumors.	Yes, is one of the reason that many urologist favor this technique	Urosepsis is the most feared complication. Frequent complications include urinary retention, hematuria, haemospermia
TRUS 10–12 cores	10–12	+	–	Easiest to perform	+	–
TRUS saturation biopsies	>20	+	–	Longer to perform / No added csPCA detection benefit	+	–
**Transperineal**				Less complications, increased diagnostic abilities (apex, anterior) / Template biopsy usually require specific equipment (brachytherapy grid, ultrasound arm) and general anesthesia. Longer procedure. Higher cost.	Most reported techniques are performed using general anesthesia. Technique with reduced core numbers can be performed using local anesthesia.	Retention is the most feared complication. Frequent complications include hematuria, haemospermia.
10-sector / 12-cores / MUSIC template	12–20	NA	NA	Can be performed using Freehand technique, avoids the cost of additional material (except for longitudinal transrectal ultrasound probe)	+	
TTPM / Barzell	~ 60 (40–160) – depending on size	+	+	Provides a 95% accurate estimation of the cancer burden / High number of cores	–	+
Ginsburg	24–38 – depending on size	+	+	Provides a high diagnostic accuracy while considerably reducing the core numbers compared to TTPM	–	+
**MRI-TB**						MRI-TB alone provides a 88–90% csPCA detection. Invisible or contralateral tumors can be missed in absence of systematic biopsies.
COG-TB, FUS-TB, IB-TB	3–5/target	–	+	No improved benefit of FUS-TB over COG-TB, or IB-TB over FUS-TB, but higher csPCA detection with IB-TB compared to COG-TB	+	+
Regional saturation	3–5/target + ~ 6 perilesional biopsies	–	+	Slightly increased csPCA detection compared to MRI-TB alone. Important role in focal therapy to assess lesion extension. Accounts for the penumbra phenomenon and possible targeting errors / No contralateral assessment	+	+

Table 1 displays pros and conn of biopsy techniques discussed in the review.

*ciPCA* clinically insignificant prostate cancer, *csPCA* clinically significant prostate cancer, *COG-TB* cognitive targeted biopsy, *FUS-TB:* *IB-TB* In-bore MRI targeted biopsy, MRI-US fusion targeted biopsy, *MRI-TB* magnetic resonance imaging targeted biopsies, *MUSIC* Michigan Urological Surgery Improvement Collaborative, *TRUS* transrectal ultrasound, *TTPM* transperineal template prostate-mapping.

## mpMRI targeted biopsies

### Key concepts

The European Association of Urology (EAU) and the American Urology Association (AUA) both integrate the use of mpMRI in their diagnostic pathway. The EAU 2024 update recommends the use of MRI-TB and perilesional prostate biopsy for patients presenting a positive mpMRI [7]. The AUA considers mpMRI as an optional exam before initial prostate biopsy, and if positive, recommend targeted biopsies (at least 2 cores) ± systematic biopsies [8].

The PI-RADS latest version (2.1) was published in 2019 to standardize image acquisition and quality to increase and spread diagnostic performance beyond reference centers [9]. The PI-QUAL score has been described as a tool to help assess mpMRI quality underlining the need for good quality images [10, 11]. Current guidelines recommend MRI-TB in the presence of equivocal or more suspicious lesions (PI-RADS ≥ 3), defined as a positive mpMRI. Because of the relatively low proportion of csPCA in the PI-RADS 3 (around 12%), current recommendation suggest using PSA-density cut-offs to decide whether to biopsy or not [12]. This is a more recent practice since most studies reported in this manuscript regarding MRI-TB usually considered PI-RADS3 as a positive mpMRI and prompted subsequent MRI-TB.

mpMRI has a 92% sensitivity to identify the index lesion, also called region of interest (ROI). The index lesion has been defined as the largest, highest grade or lesion with extraprostatic extension (EPE). The index lesion is thought to be the lesion containing the most aggressive cancer cells, and therefore the clone with metastasis potential. Treating this lesion in patients in whom it can be identified is therefore the aim of focal therapy [13, 14]. While mpMRI is a central tool in the MRI-TB pathway, it tends to underestimate the actual burden of prostate cancer, as well as its borders, that are usually irregular and not as circumscribed as the mpMRI lesion [15, 16].

### Fusion biopsies : techniques, accuracy and comparison

The benefits of MRI-based diagnostics heavily depend on image quality and reading quality [17, 18]. The region of interest (ROI) on MRI has to be identified and marked by the specialist reader and then be transferred onto the live prostate during the biopsy. The ROI information can be used with cognitive transfer, software-driven US-MRI fusion and in-bore targeting. Cognitive targeted biopsy (COG-TB) describes a term where the operator uses his brain only to register MRI-derived targets to a transrectal ultrasound. No additional equipment to standard transrectal ultrasound is required, hence it is the easiest form with no cost implication of fusion [19]. However, it inevitably carries a risk of operator-dependent transfer error. MRI-US fusion targeted biopsy (FUS-TB) consists of a fusion of previously acquired mpMRI images with pre-marked ROI with real-time TRUS imaging, allowing for a potentially more precise targeting. Several platforms have been successfully developed, with no clear superior diagnostic ability reported of one over the others [20]. In-bore MRI targeted biopsy (IB-TB) implies to proceed to the biopsy directly in the bore of an MRI scanner, requiring specific compatible equipment. It usually requires the previous acquisition of mpMRI images to identify lesions, and a reacquisition at the moment of biopsy. Usually, a sedation or general anesthesia is performed, while only targeted-biopsies are acquired because of the lengthy time and difficulty to perform systematic biopsies at the same time [21].

When comparing the three different techniques of targeting techniques in a systematic review, Wegelin et al. could show no difference in 2019 between FUS-TB and COG-TB (sensitivity of 81% vs. 72%, *p* = 0.11), while IB-TB significantly outperformed COG-TB (sensitivity 89% vs. 72%, *p* = 0.02) for PCA detection. For csPCA, COG-TB had an 86% sensitivity similar to FUS-TB (89%, *p* = 0.62) and MRI-TB (92%, *p* = 0.42). In this meta-analysis encompassing records utill 2015, 43 studies were included, 39.5% (17/43) using FUS-TB, 25.6% (11/43) using IB-TB, and 25.6% (11/43) using COG-TB. In 2022, Bass et al. reported an updated systematic review and showed that FUS-TB was the most represented technique (76.7%; 33/43 studies) while COG-TB (18.6%; 8/43) came second and IB-TB came last (4.6%; 2/43), possibly suggesting a decreased used of IB-TB with the larger implantation of FUS-TB [22]. This meta-analysis confirmed similar sensitivities of IB-TB (87%), COG-TB (81%) and FUS-TB (81%) for the detection of csPCA (*p* = 0.55), as well as similar ciPCA yield of IB-TB (10%), COG-TB (5%) and FUS-TB (8%, *p* = 0.46). Wegelin et al. afterwards reported the results of the FUTURE trial, a multicenter randomized control trial that confirmed the absence of difference regarding csPCA between the different MRI-TB techniques. They however stated as an important limitation the lack of power of the study, that would have required the inclusion of 9886 additional patients [23].

When comparing MRI-TB to transrectal ultrasound guided systematic biopsy (TRUS-SB), both meta-analyses agreed ; Wegelin reported an increased detection of csPCA using MRI-TB (RR : 1.16) corresponding to an MRI-TB sensitivity of 90% compared to TRUS-SB sensitivity of 79%, as well as a decreased detection of ciPCA (RR = 0.47). Bass confirmed these findings, with an increased detection of csPCA seen with MRI-TB (RR : 1.24, *p* = 0.02) compared to TRUS-SB corresponding to an MRI-TB sensitivity of 83% compared to TRUS sensitivity of 63%, as well as a decreased detection of ciPCA (RR = 0.58, *p* < 0.01). Overall, data on fusion biopsies can be biased by differences in urologist expertise. However, trained residents ( > 50 cases) tend to perform similar as consultant urologists, suggesting an acceptable learning curve [24]. Microultrasound, is a novel technology using a high-frequency 29-Mhz transrectal probe, that allows real-time recognition and targeting of lesions during biopsy. The diagnostic value of this technology seems to be comparable to MRI, at least in expert centers [25]. In addition, MRI and microultrasound seem to be complementary technologies that might enhance the risk stratification, especially in case of focal therapy [26]. An explanatory RCT evaluating microultrasound against MRI in ongoing, and will further clarify the role of this technology [27]. Another promising technology is the multiparametric ultrasound (mpUS). Recently, a high-quality diagnostic study (CADMUS) has compared mpUS to mpMRI and showed a 73% agreement between both modalities. Each test alone resulted in a 26% vs. 30% csPCA detection rate respectively for patient that underwent modality diagnosed and guided biopsies. While mpUS detected slighty less csPCA (−4%), it also increased the number of patients referred for biopsies, still being inferior to mpMRI [28]. Further studies are required to precise its future perspectives in prostate cancer diagnosis, but it could already play an interesting role for patient that cannot benefit from mpMRI.

### Role of perilesional sampling

Several authors reported on the importance of perilesional sampling [29, 30]. Brisbane et al. accounted for the phenomenon of invisible cancer around the index lesion, under the concept of “penumbra” ; a distance starting at the border of the ROI and containing the 90% of all csPCA in the gland. They showed that only 50% of the csPCA was contained within the ROI for patients presenting with PI-RADS 3, compared to 60% for PI-RADS 4 and 74% for PI-RADS 5. The radius of the penumbra depended on the PI-RADS score, with an additional perimeter of 16 mm, 12 mm and 5 mm for PI-RADS 3, 4 and 5 respectively [29]. This concept is important in focal therapy since margins of treatment have to been assessed [31, 32].

Meanwhile, Hansen et al. established in 2020 that a targeted saturation biopsy on the same side as the ROI is highly effective for diagnosing cancer, although the exact size of the “penumbra” relative to the PIRADS score of the ROI remains a subject of discussion [32]. More recently, Noujeim et al. conducted a thorough analysis to assess the distance between systematic cores containing clinically significant prostate cancer (csPCa) and the region of interest (ROI). The authors demonstrated that sampling around the lesion combined with TB was as effective as SB + TB for detecting csPCa (35% vs. 37%, *p* = 0.2). By employing a machine learning algorithm and categorizing three risk groups based on PIRADS score and PSA density, they found the risk of missing csPCa beyond the 10 mm penumbra was 2%, 8%, and 29% for low, intermediate, and high-risk groups, respectively [33]. Furthermore, the cumulative distribution rate for csPCa reached 86% within a 10 mm margin. It was concluded that for men with PIRADS 3–5 lesions and a PSA density below 0.15 ng/ml^2^, biopsies beyond the 10 mm penumbra might be unnecessary. This goes in the same direction as the 2019 PIRADS committee recommendation to conduct template biopsies (TB) of the region of interest (ROI) in addition with a 5-mm penumbra for lesions rated as PIRADS 4 and 5 [34]. Tafuri et al. also noted that for PIRADS 5 lesions with a PSA density greater than 0.15 ng/ml^2^, systematic samples offered only a marginal increase in csPCA detection [35]. Standard biopsy seems to remain important, especially in the perilesional area, especially for PI-RADS 4/5 lesions and a PSA density above 0.15 ng/ml. If confirmed in wider studies, these findings suggest the need for a risk-group based approach, based on PI-RADS score and PSA density to potentially avoid systematic or contralateral biopsies [36–38].

## Systematic biopsies

While systematic transrectal biopsies were a long-lasting standard, their role has been superseded by mpMRI targeted as seen in the previous section. Delongchamps et al. reported the result of a per-patient analysis comparing TRUS-SB (10–12 cores) to TRUS-TB (3 cores). They reported a reduced overall PCA rate but a similar csPCA rate (46.2% vs. 48.1%, *p* = 0.69) [39]. Later, the PRECISION trial showed a higher detection of csPCA in biopsy-naïve patients benefitting from transrectal (TR) MRI-TB (38% vs. 26%) compared to TRUS-SB alone, while 28% of the randomized men in the MRI group could avoid biopsies [4]. Despite this best performance, allowing for a precise staging with less cores, about between 0 to 16% csPCA would be missed if concomitant systematic biopsies were not performed [39–42].

**Fig. 2:**
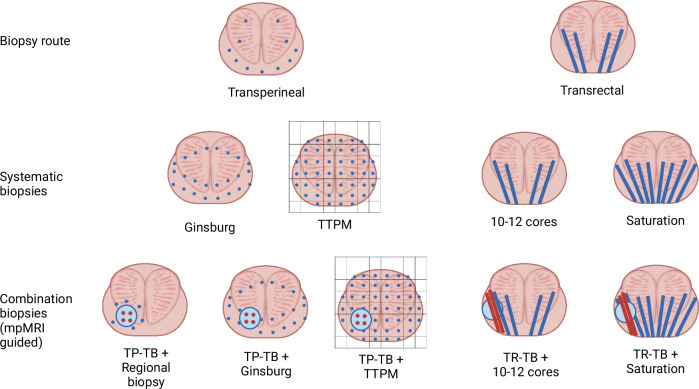
Frequently employed prostate biopsy techniques. Figure 2 displays the most frequently employed biopsy techniques, differentiated by the biopsy route, type of systematic biopsies and use of targeted biopsies. It displays a coronal cut of the prostate and the relative positive of the biopsies. Transperineal biopsies are perpendicular to the coronal plane and shown as dots, whereas transrectal are parallel and appear as traits. Ginsburg protocol uses anterior, mid-sector and posterior biopsies (as displayed) and depends on the size of the gland (this template is valid for glands up to 30 ml). For glands larger than 30 ml, 8 additional biopsies (not displayed on this figure) are obtained from the basal sector, in the extension of the mid-sector (total 32 cores). For glands larger than 50 ml, the number of cores per sector (anterior, mid and posterior) and per side is 5 (total 38 cores). TTPM uses a brachytherapy grid to obtain biopsies at regular interval of 5 mm. Created with Biorender.com. TP transperineal, TR transrectal, TB targeted biopsy, TTPM transperineal template prostate-mapping.

Systematic samples of the prostate might be performed in different ways. These techniques are summarized as well in Fig. 2.

### TRUS 10–12 systematic cores (TRUS-SB)

10 or 12-TRUS cores was considered the standard TRUS-guided technique and its technique has been largely reported [43]. The main problem that arose from 10–12 TRUS cores pattern is the random and systematic errors. Random errors are due to the absence of targeting ; it promotes the detection of ciPCA while missing csPCA. Systematic errors happen when a zone (such as the anterior zone, anterior zones and midline under the urethra) is hard to target and will systematically be missed or inadequately sampled [44]. Therefore, the TRUS-SB present with a high risk of false negative (30–45%) and an accuracy of only around 59% [41, 44]. Valerio et al. compared the efficiency (number of cores required to detect one significant cancer) and showed that 37 cores vs. 9 cores were required respectively for TRUS-SB compared to MRI-TB (median difference of 32.1 cores). When assessing utility (number of men with PCA that have been detected with a sampling strategy that would have been missed using the other strategy), they reported an 9% additional csPCA detection using MRI-TB, compared to 2% when using TRUS-SB alone [45].

### TRUS saturation biopsies (TRUS-SatB)

The term “saturation biopsies” (SatB) has been reported several times in the literature, but no clear and accepted definition defines it. Most authors agree that it is a higher density biopsy protocol, that usually includes at least 20 cores. It has mainly been advocated for re-biopsy after one or several previous negative TRUS-SB, before MRI-TB was recommended, since it allowed the detection of previously missed csPCA [46, 47]. Despite initial promising results, a randomized control trial showed no benefit when comparing 20-cores to the standard 12-cores biopsies, therefore limiting its widespread adoption afterwards [2, 47, 48].

### Transperineal systematic biopsies

**Fig. 3:**
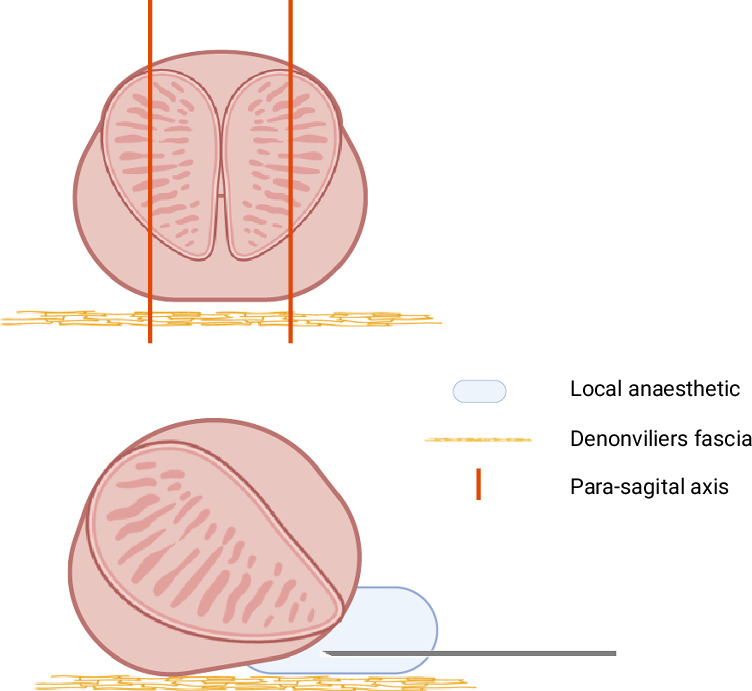
Transperineal local anesthesia technique. Figure 3 displays one of the frequently used and reported technique (Emiliozzi). The orange line illustrate the para-sagital axis where the puncture will be made. This axis is found using a 45 degree angle from the anus in the lithotomy position, at a 1.5 cm distance on each side (left and right). Using a linear transrectal ultrasound probe, the sagittal (more precisely the para-sagital axis) is obtained. A needle (22 G) is inserted between the Denonviliers fascia and the apex of the prostate. Slow injection will lift the prostate upwards and be visible. The technique requires a bilateral injection of 10 ml of rapid acting anesthetic [83]. Created with Biorender.com.

The first description of the technique of transperineal, ultrasound guided biopsies was reported in 1981 by Holm and Gammelgard [49]. Based on the transrectal techniques, several templates were reported. The most known and standardized techniques are the Transperineal Template Prostate Mapping (TTPM), and Ginsburg protocol. Other techniques have been reported, such as the 10-sector template, 12-core template and the MUSIC template [50]. These techniques, using a reduced number of cores, are possible to perform under local anesthesia. Figure 3 provides an illustration of the local anesthesia for TP-Bx.

The concept of TTPM was first described by Barzell and Whitmore in 2003 and later standardized in 2007 [51]. TTPM uses a brachytherapy grid to exhaustively biopsy the prostate, with cores taken every 5 mm, with at least one biopsy from each hole of the grid [52]. On a 3-dimensional virtually created model, this strategy yields a > 95% correct risk stratification of all clinically significant cancers (defined as lesions of 0.5 ml or greater) [53]. This strategy results in a high biopsy density, and high number of cores per patient (mean of 63 cores, ranges described as high as 160 for very large glands) [53]. Valerio et al. reported that reducing the number of biopsies has a negative impact on its ability to exclude clinically significant disease and is therefore inferior [54]. TTPM served as a the gold-standard for the PROMIS study that was the first level 1b evidence-study to prove the increased sensitivity of MRI-TB compared to TRUS-SB [5].

The Ginsburg protocol is based on a multidisciplinary panel agreement (Ginsburg Study Group for Enhanced Prostate Diagnostics) to provide a reproducible dataset to standardize reporting among future studies. The number of cores is directly dependent on the prostate size ; it involves a minimum of 24 cores for prostate **≤**30 ml (as displayed in Fig. 2), 32 cores for prostate >30–50 ml and length >4 cm, and up to 38 cores for larger prostate [52]. After its original description, it was tested on 534 patients with a median number of biopsy cores of 26 (IQR 24–28) and a mean procedure length between 25 to 60 min. It allowed the detection of csPCA in 39% biopsy-naïve patients, 27% of patients that previously had previous negative biopsies, and 45% of patients on active surveillance for low-risk cancers [55].

## Combined targeted and systematic biopsies

Combined MRI-TB and TRUS-SB or TP systematic biopsies (TP-SB) is recommended as the upfront strategy for biopsy-naïve men by the European Association of Urology [6]. In general, MRI-TB consist of 3–4 cores/target, but more variations exist regarding SB, also depending on the route used. In general, ipsilateral and contralateral biopsies to MRI-TB are collected. 10–12 cores (TR or TP), Ginsburg protocol, TTPM or RB have been described in addition to MRI-TB.

Hagens et al. conducted a recent meta-analysis comparing the performance of MRI-TB alone compared to MRI-TB + RB or MRI-TB + SB. The median number of cores was 9.5 in the MRI-TB + RB group compared to 16.5 in the MRI-TB + SB, while no difference in csPCA detection was seen (RR = 0.95, *p* = 0.09). Interestingly, the RB strategy avoided contralateral SB. As awaited, MRI-TB + SBx overperformed when compared to MRI-TB only (RR:1.24, *p* < 0.001), but at a cost of increasing the median number of cores from 3.5 to 16.5. Interestingly, when adding RB to MRI-TB, only 6 cores were necessary to increase substantially the detection compared to MRI-TB alone (RR:1.18, *p* < 0.001) [56]. The risk of such strategy is however a grade inflation and grade shift that may overestimate the cancer burden [57]. This grade shift may compromise the use of nomograms, frequently used for lymph node invasion or EPE prediction. Another recent prospective RCT compared MRI-TB and regional saturation biopsies (RSB) using 9 cores, for men with PSA 4–20 ng/ml, and found a similar performance of MRI + RSB compared to MRI-TB + SB (csPCA detection rate in 44.1% vs. 40.7%, *p* = 0.3) while requiring less cores. It significantly overperformed TB alone (csPCA detection rate 44.1% vs. 31.8, *p* = 0.01). When comparing their biopsy results with whole mount histology (WMH) analysis, 97% of the significant cancer were identified using RSB. They could also determine that the average underestimation of size comparing WMH and mpMRI was 0.76 cm confirming previously reported mpMRI cancer burden underestimation of 0.9 cm suggested by Le Nobin et al. [15, 31]. Tschirdewahn et al. reported a per-patient detection of 99% of csPCA using the RB-saturation technique compared to 87% (*p* = 0.001) for MRI-TB (4 cores) and 81% (*p* < 0.001) for extended systematic biopsies (24 cores), suggesting increased diagnostic performance of the RB-saturation technique [58]. However, the same team conducted afterwards a RCT comparing 4 cores MRI-TB to MRI-TB + RB (9 cores total) where they could not show a statistical difference regarding per-patient csPCA detection (91.6% vs. 100%, *p* = 0.058) [59]. Although these results did not show a statistical difference, they raise the question whether the Ginsburg protocol should still be adopted, or if MRI-TB + RB may be accurate enough to supersede it [32].

## Transrectal vs. transperineal route

Some factors have been limiting the widespread adoption of TP. The need for general anesthesia at the beginning of the experience, and the need to switch equipment for a generation of urologists naïve to the TP approach have limited initial widespread adoption. Also, the biopsy strategy needs to take into account external factors as well, such as the time of the procedure, cost and access to the operating theater, since TP are usually more expensive, lengthy and less frequently performed under local anesthesia [39, 60, 61]. For example, Altok et al. reported in a cost-efficacy analysis a 2.5x times increase of the price of general anesthesia for TTPM (3554$) compared to local anesthesia TRUS-SB (1405$), with a cost even superior to in-bore MRI biopsy (2.2x times increase – 3158 $) [61]. Also, until recently, there was no certainty regarding the staging accuracy of TP route compared to TR route.

Ber et al. reported a non-inferiority within-person study comparing TP and TR, that proved non-inferiority and actually suggested that TP route was superior for csPCA detection rate (42% vs. 27%, *p* = 0.03) [62]. Loy et al. found a comparable sensitivity and specificity (81% vs. 80%, 99% vs. 95%, for TR and TP respectively) in a recent systematic analysis and meta-analysis [63]. A large recent multicenter retrospective cohort including 5200 patients compared TR and TP route for patients benefitting of MRI-TB for lesions PI-RADS ≥ 3 and showed a higher rate of PCA and csPCA detection using TP route (64% vs. 50%, 49% vs. 35%, *p* < 0.01 respectively) [64]. TP route was an independent predictor of PCA (OR:1.37) and especially csPCA (OR:1.19), with higher performance in the apex (OR:4.81), transition/central zone (OR:2.67) and anterior zone (OR:5.62). Those finding were confirmed by Uleri et al. in a 2023 systematic review and meta-analysis including 8662 patients, showing that TP significantly outperformed TR for anterior lesions (OR : 2.17, *p* < 0.001) and apical lesions (OR : 1.86, *p* = 0.01) with a similar overall csPCA rate (OR = 1.11, *p* = 0.1). TP significantly outperformed TR for PI-RADS 4 lesions (OR : 1.57, *p* = 0.02), possibly hinting at a more precise targeting with TP since PI-RADS 4 lesions are usually smaller [65]. Wu et al. also showed a higher csPCA detection rate of TP compared to TR (RR 1.33, *p* = 0.005) on a per-patient analysis and comparing two cohorts (RR:1.37, *p* = 0.0002) as well. They also reported an increased csPCA detection in the anterior region on a per patient analysis (RR:2.55) as well as per-lesion analysis (RR:1.52), but did not provide information regarding apical lesions [66]. These findings confirm that TP is an adequate and safe substitute to transrectal mpMRI targeted lesions, and possibly provides a higher diagnostic accuracy when focusing on apical and anterior lesions that are subject to systematic sampling error using the TR route.

## Safety aspects

Another important aspect to discuss is the possible complications related to transperineal and transrectal route. Overall, some trials have assessed the complications of the TP + antibiotics, TP alone and TR + antibiotics strategies. A systematic review (SR) including 165 studies with 162,577 patients reported sepsis rates of 0.1% for TP and 0.9% for TR biopsies [67]. Additionally, a population-based study from the UK involving 73,630 patients demonstrated lower re-admission rates for sepsis in those who underwent TP biopsies (1.0%) compared to those who underwent transrectal biopsies (1.4%) [68]. These results collectively indicate a lower risk of infectious complications with transperineal biopsies compared to transrectal methods. However, a recent randomized controlled trial by Mian et al. of 840 men challenges the abovementioned results. In terms of complications both routes showed similar results with a 2,6% and 2,7% of infectious complications and a 1,7% and 2,2% rate of other complications [69]. The present study with such large numbers clearly challenges the results of the Cochrane review. Regarding the u antibiotic prophylaxis, a multicenter, randomized trial compared TP biopsy without antibiotic prophylaxis to TR biopsy with targeted prophylaxis based on rectal culture screening. No infections were reported in the TP group, whereas the TR group experienced a 1.4% infection rate (difference –1.4%; *p* = 0.059). Participants undergoing TP biopsies reported higher levels of periprocedural pain, with a small adjusted difference of 0.6 on a 0–10 scale, but this discomfort resolved within 7 days [70]. A systematic review (SR) and meta-analysis of eight non-randomized controlled trials (non-RCTs) evaluated the impact of antibiotic prophylaxis on infection outcomes for patients undergoing TP biopsies. The analysis found no significant differences between patients who received antibiotic prophylaxis and those who did not in terms of post-biopsy infection rates (0.11% vs. 0.31%) and sepsis rates (0.13% vs. 0.09%) [71]. These results suggest that antibiotic prophylaxis may not significantly affect the incidence of infections or sepsis following TP biopsies, which might be another reason to choose TP over TR route.

## Focal therapy : adaptation of the biopsy strategy

After having described several prostate biopsy strategies, the question is ; is there a recognized “best strategy” for focal therapy? The Ginsburg Protocol and TTPM have been advocated as a reference for focal therapy, but required to be performed under general anesthesia which is a non-deliverable expensive strategy across the board. Also, minimizing the toxicity is also important, since higher core number is associated with increased adverse events [72]. To answer this question, several authors have compared the sensitivity of several biopsy strategy to WMH to identify whether preoperative biopsy were accurate enough to detect all csPCA.

Nassiri et al. investigated FT eligibility of men that underwent MRI-TB using FUS-TB and 12-cores TRUS-SB. On a cohort of 454 men with biopsy proven region of interest, 175 were candidates after biopsies. When compared with WHM, this biopsy strategy showed a 80% sensitivity, 73.5% specificity, 75% accuracy for FT eligibility [73]. Johnson et al. investigated the reliability of MRI-TB using TR route. They included 665 patients of a prospectively maintained database and identified 92 as candidates after the biopsies. Among them, 44 (48%) were inadequately considered as candidate when studying the WMH. Reason for exclusion was the tumor crossing the midline (21 patients), contralateral csPCA (20 patients) and ipsilateral high-risk tumor (3 patients). Interestingly, men with anterior index tumor where 2.4 times more likely to present undetected contralateral csPCA when compared to men with posterior tumor. Unfortunately, the number of targeted cores and the total number of cores were not reported [74]. Choi reported in 2023 a study on 120 men that underwent TTPM + MRI-TB and RP. On the 120 studied men, 52 were deemed eligible. Forty-two (80.7%) of them were correctly eligible based on WMH, while only 10 (19.2%) would have been contra-indicated (6 had bilateral disease, 4 had small ISUP2 volume priorly undetected), suggesting a more accurate staging. Their biopsy strategy used a median 29 cores on mean prostatic volume of 36 ml and required general anesthesia [75]. Lee recently reported a retrospective analysis of 398 patients that underwent TP-SatB (around 24 cores) combined with TP MRI-TB. They studied the impact of the reduction of number of systematic cores on the eligibility for focal therapy, using 4 different strategies (2/3 cores, ½ cores, 1/3 cores, ¼ cores), using a computer algorithm to evenly and artificially suppress biopsies and erase any systematic biopsy that overlapped MRI-TB. Patients had a treatment plan based on the biopsy finding, that could either consist of single quadrant ablation, hemi-ablation (anterior or lateral), three-quadrant ablation or whole-gland ablation. They reported a median number of 33 cores per patient [28–39], with a median of 9 targeted cores. When compared to the plan that would have been realized on the basis of MRI-TB alone, adding the full systematic biopsy set resulted in a treatment plan change in 44% of the patients, with 10% of them becoming unsuitable for FT. Reducing the number of systematic cores had an important impact on FT treatment plan, with a modification in 12% of the patient (2/3 cores), 19% (1/2 cores), 24% (1/3 cores), and 29% (1/4 cores), showing that inadequate systematic sampling would probably result in suboptimal focal therapy planning [40].

Several consensus statement have been published for preoperative biopsy strategy as well as follow-up. Ong et al. studied all consensuses statement published regarding focal therapy up to 2023. All these consensuses agreed that mpMRI was the imaging modality of choice, and that MRI-TB + SB were required. While some authors agreed that in absence of mpMRI the TTPM can be sufficient, other agreed that systematic TRUS biopsies were sufficient, underlining the ongoing heterogeneity of practice and lack of identified “best biopsy strategy” yet [76]. The heterogeneity in csPCa definition as well as what we can accept as a community in the untreated area of men undergoing focal therapy are matters of debate which widely explain the ongoing controversy. This is beyond the purpose of this study. Follow-up after focal therapy is needed since as high as 20–30% patients will require re-treatment after the 1^st^ treatment. While no consensus was reached, most consensuses recommend a first mpMRI at 6 month with follow-up prostate biopsy at 6–12 months (targeted on the treated region, and SB) [76].

## Conclusion

A large heterogeneity of practice exists regarding prostate biopsies, in term of access (transrectal versus transperineal), type of targeting used for MRI-targeted biopsies (cognitive, software-based, in-bore), and template of systematic biopsies. Transrectal access is easier to perform under local anesthesia and is still widely perform. Transperineal has been advocated as a safer route because of a reduced risk of postoperative urosepsis, possibility to perform without antibiotic prophylaxis, and increasing evidence show increased diagnostic performance especially in the apex and anterior zone, that are difficult zones to biopsy using transrectal biopsy. Biopsy protocols have emerged to standardize study reporting concerning transperineal biopsies, especially for focal therapy but have not seen widespread adoption yet. Systematic biopsies are subject to random and systematic errors and often misclassify patient (overdetection of ciPCA, underdetection of csPCA) and have been progressively abandoned as an alone procedure. MRI-TB alone reduces the detection of ciPCA but misses csPCA when compared to combined biopsy techniques, therefore asking for adequate combination strategy. Among the combinations and systematic strategies, the TTPM and Ginsburg protocol have shown to be have the highest negative predictive value, but require a high number of cores, exposing patients to potential combinations and the need for general anesthesia that limit this widespread implantation. Regional biopsies are gaining a lot of interest recently, since they have an overall sensitivity of about 90%, while importantly reducing the number of cores and avoiding the detection of ciPCA and are easily performed under local anesthesia. PSA density, and PI-RADS score as important markers to guide the biopsy strategy (only ipsilateral biopsies (regional)) or addition of contralateral biopsies. Finally, the accuracy of staging has a direct impact on focal therapy eligibility, with the need to define the borders of the index lesion to target as well as to minimize the likelihood of missed csPCA that may expose the patient to undertreatment. All these aspects have to be taken into consideration when planning biopsy for patients who might be eligible and considering focal therapy.

## References

[1] Hodge KK, McNeal JE, Terris MK, Stamey TA. Random systematic versus directed ultrasound guided transrectal core biopsies of the prostate. J Urol. 1989;142:71–4. 2659827 10.1016/s0022-5347(17)38664-0

[2] Ukimura O, Coleman JA, de la Taille A, Emberton M, Epstein JI, Freedland SJ, et al. Contemporary role of systematic prostate biopsies: indications, techniques, and implications for patient care. Eur Urol. 2013;63:214–30. 23021971 10.1016/j.eururo.2012.09.033

[3] Liu W, Patil D, Howard DH, Moore RH, Wang H, Sanda MG, et al. Adoption of prebiopsy magnetic resonance imaging for men undergoing prostate biopsy in the United States. Urology. 2018;117:57–63. 29679601 10.1016/j.urology.2018.04.007

[4] Kasivisvanathan V, Rannikko AS, Borghi M, Panebianco V, Mynderse LA, Vaarala MH, et al. MRI-targeted or standard biopsy for prostate-cancer diagnosis. N. Engl J Med. 2018;378:1767–77. 29552975 10.1056/NEJMoa1801993 PMC9084630

[5] Ahmed HU, El-Shater Bosaily A, Brown LC, Gabe R, Kaplan R, Parmar MK, et al. Diagnostic accuracy of multi-parametric MRI and TRUS biopsy in prostate cancer (PROMIS): a paired validating confirmatory study. Lancet. 2017;389:815–22. 28110982 10.1016/S0140-6736(16)32401-1

[6] Mottet N, van den Bergh RCN, Briers E, Van den Broeck T, Cumberbatch MG, De Santis M, et al. EAU-EANM-ESTRO-ESUR-SIOG guidelines on prostate cancer-2020 update. Part 1: screening, diagnosis, and local treatment with curative intent. Eur Urol. 2021;79:243–62. 33172724 10.1016/j.eururo.2020.09.042

[7] Cornford P, van den Bergh RCN, Briers E, Van den Broeck T, Brunckhorst O, Darraugh J, et al. EAU-EANM-ESTRO-ESUR-ISUP-SIOG guidelines on prostate cancer—2024 update. Part i: screening, diagnosis, and local treatment with curative intent. Eur Urol. 2024; Available from: https://www.sciencedirect.com/science/article/pii/S0302283824022541. 10.1016/j.eururo.2024.03.027 38614820

[8] Wei JT, Barocas D, Carlsson S, Coakley F, Eggener S, Etzioni R, et al. Early detection of prostate cancer: AUA/SUO guideline part i: prostate cancer screening. J Urol. 2023; Available from: 10.1097/JU.0000000000003491. 10.1097/JU.0000000000003491 PMC11060750 37096582

[9] Turkbey B, Rosenkrantz AB, Haider MA, Padhani AR, Villeirs G, Macura KJ, et al. Prostate imaging reporting and data system version 2.1: 2019 update of prostate imaging reporting and data system version 2. Eur Urol. 2019;76:340–51. 30898406 10.1016/j.eururo.2019.02.033

[10] Giganti F, Allen C, Emberton M, Moore CM, Kasivisvanathan V. PRECISION study group. Prostate imaging quality (PI-QUAL): a new quality control scoring system for multiparametric magnetic resonance imaging of the prostate from the PRECISION trial. Eur Urol Oncol. 2020;3:615–9. 32646850 10.1016/j.euo.2020.06.007

[11] Windisch O, Benamran D, Dariane C, Favre MM, Djouhri M, Chevalier M, et al. Role of the prostate imaging quality PI-QUAL score for prostate magnetic resonance image quality in pathological upstaging after radical prostatectomy: a multicentre European study. Eur Urol Open Sci. 2023;47:94–101. 36601048 10.1016/j.euros.2022.11.013 PMC9806708

[12] Uroweb - European Association of Urology [Internet]. Citing, Usage & Republication - Uroweb. 2024 Available from: https://uroweb.org/eau-guidelines/citing-usage-republication.

[13] Liu W, Laitinen S, Khan S, Vihinen M, Kowalski J, Yu G, et al. Copy number analysis indicates monoclonal origin of lethal metastatic prostate cancer. Nat Med. 2009;15:559–65. 19363497 10.1038/nm.1944 PMC2839160

[14] Ahmed HU. The index lesion and the origin of prostate cancer. N Engl J Med. 2009;361:1704–6. 19846858 10.1056/NEJMcibr0905562

[15] Le Nobin J, Rosenkrantz AB, Villers A, Orczyk C, Deng FM, Melamed J, et al. Image guided focal therapy for magnetic resonance imaging visible prostate cancer: defining a 3-dimensional treatment margin based on magnetic resonance imaging histology co-registration analysis. J Urol. 2015;194:364–70. 25711199 10.1016/j.juro.2015.02.080 PMC4726648

[16] Priester A, Natarajan S, Khoshnoodi P, Margolis DJ, Raman SS, Reiter RE, et al. Magnetic resonance imaging underestimation of prostate cancer geometry: use of patient specific molds to correlate images with whole mount pathology. J Urol. 2017;197:320–6. 27484386 10.1016/j.juro.2016.07.084 PMC5540646

[17] Gaziev G, Wadhwa K, Barrett T, Koo BC, Gallagher FA, Serrao E, et al. Defining the learning curve for multiparametric magnetic resonance imaging (MRI) of the prostate using MRI-transrectal ultrasonography (TRUS) fusion-guided transperineal prostate biopsies as a validation tool. BJU Int. 2016;117:80–6. 25099182 10.1111/bju.12892

[18] Brizmohun Appayya M, Adshead J, Ahmed HU, Allen C, Bainbridge A, Barrett T, et al. National implementation of multi-parametric magnetic resonance imaging for prostate cancer detection – recommendations from a UK consensus meeting. BJU Int. 2018;122:13–25. 29699001 10.1111/bju.14361 PMC6334741

[19] Rifkin MD, Zerhouni EA, Gatsonis CA, Quint LE, Paushter DM, Epstein JI, et al. Comparison of magnetic resonance imaging and ultrasonography in staging early prostate cancer. N. Engl J Med. 1990;323:621–6. 2200965 10.1056/NEJM199009063231001

[20] Paesano N, Catalá V, Tcholakian L, Trilla E, Morote J. A systematic review of the current status of magnetic resonance-ultrasound images fusion software platforms for transperineal prostate biopsies. Cancers. 2023;15:3329. 37444439 10.3390/cancers15133329 PMC10340408

[21] Le JD, Huang J, Marks LS. Targeted prostate biopsy: value of multiparametric magnetic resonance imaging in detection of localized cancer. Asian J Androl. 2014;16:522–9. 24589455 10.4103/1008-682X.122864 PMC4104074

[22] Bass EJ, Pantovic A, Connor MJ, Loeb S, Rastinehad AR, Winkler M, et al. Diagnostic accuracy of magnetic resonance imaging targeted biopsy techniques compared to transrectal ultrasound guided biopsy of the prostate: a systematic review and meta-analysis. Prostate Cancer Prostatic Dis. 2022;25:174–9. 34548624 10.1038/s41391-021-00449-7 PMC9184263

[23] Wegelin O, Exterkate L, van der Leest M, Kummer JA, Vreuls W, de Bruin PC, et al. The FUTURE Trial: a multicenter randomised controlled trial on target biopsy techniques based on magnetic resonance imaging in the diagnosis of prostate cancer in patients with prior negative biopsies. Eur Urol. 2019;75:582–90. 30522912 10.1016/j.eururo.2018.11.040

[24] Turchi B, Lombardo R, Franco A, Tema G, Nacchia A, Cicione A, et al. Residents and consultants have equal outcomes when performing transrectal fusion biopsies: a randomized clinical trial. Curr Oncol. 2024;31:747–58. 38392049 10.3390/curroncol31020055 PMC10887997

[25] Sountoulides P, Pyrgidis N, Polyzos SA, Mykoniatis I, Asouhidou E, Papatsoris A, et al. Micro-ultrasound-guided vs. multiparametric magnetic resonance imaging-targeted biopsy in the detection of prostate cancer: a systematic review and meta-analysis. J Urol. 2021;205:1254–62. 33577367 10.1097/JU.0000000000001639

[26] Rakauskas A, Peters M, Martel P, van Rossum PSN, La Rosa S, Meuwly JY, et al. Do cancer detection rates differ between transperineal and transrectal micro-ultrasound mpMRI-fusion-targeted prostate biopsies? A propensity score-matched study. PLoS ONE. 2023;18:e0280262. 36652429 10.1371/journal.pone.0280262 PMC9847953

[27] Klotz L, Andriole G, Cash H, Cooperberg M, Crawford ED, Emberton M, et al. Optimization of prostate biopsy - Micro-Ultrasound versus MRI (OPTIMUM): A 3-arm randomized controlled trial evaluating the role of 29 MHz micro-ultrasound in guiding prostate biopsy in men with clinical suspicion of prostate cancer. Contemp Clin Trials. 2022;112:106618. 34728381 10.1016/j.cct.2021.106618

[28] Grey ADR, Scott R, Shah B, Acher P, Liyanage S, Pavlou M, et al. Multiparametric ultrasound versus multiparametric MRI to diagnose prostate cancer (CADMUS): a prospective, multicentre, paired-cohort, confirmatory study. Lancet Oncol. 2022;23:428–38. 35240084 10.1016/S1470-2045(22)00016-X

[29] Brisbane WG, Priester AM, Ballon J, Kwan L, Delfin MK, Felker ER, et al. Targeted prostate biopsy: umbra, penumbra, and value of perilesional sampling. Eur Urol. 2022;82:303–10. 35115177 10.1016/j.eururo.2022.01.008

[30] Raman AG, Sarma KV, Raman SS, Priester AM, Mirak SA, Riskin-Jones HH, et al. Optimizing spatial biopsy sampling for the detection of prostate cancer. J Urol. 2021; Available from: 10.1097/JU.0000000000001832. 10.1097/JU.0000000000001832 PMC8903239 33908801

[31] Jiang X, Chen M, Tian J, Li X, Liu R, Wang Y, et al. Comparison of regional saturation biopsy, targeted biopsy, and systematic biopsy in patients with prostate-specific antigen levels of 4–20 ng/ml: a prospective, single-center, randomized controlled trial. Eur Urol Oncol. 2024;7:944–53. 10.1016/j.euo.2023.12.002 38158249

[32] Hansen NL, Barrett T, Lloyd T, Warren A, Samel C, Bratt O, et al. Optimising the number of cores for magnetic resonance imaging-guided targeted and systematic transperineal prostate biopsy. BJU Int. 2020;125:260–9. 31306539 10.1111/bju.14865 PMC8641376

[33] Noujeim JP, Belahsen Y, Lefebvre Y, Lemort M, Deforche M, Sirtaine N, et al. Optimizing multiparametric magnetic resonance imaging-targeted biopsy and detection of clinically significant prostate cancer: the role of perilesional sampling. Prostate Cancer Prostatic Dis. 2023;26:575–80. 36509930 10.1038/s41391-022-00620-8

[34] Padhani AR, Weinreb J, Rosenkrantz AB, Villeirs G, Turkbey B, Barentsz J. Prostate imaging-reporting and data system steering committee: PI-RADS v2 status update and future directions. Eur Urol. 2019;75:385–96. 29908876 10.1016/j.eururo.2018.05.035 PMC6292742

[35] Tafuri A, Iwata A, Shakir A, Iwata T, Gupta C, Sali A, et al. Systematic biopsy of the prostate can be omitted in men with PI-RADS^TM^ 5 and prostate specific antigen density greater than 15. J Urol. 2021;206:289–97. 33818141 10.1097/JU.0000000000001766

[36] Thomas C. Perilesional sampling: the new standard for imaging-targeted prostate biopsies? Prostate Cancer Prostatic Dis. 2023;26:439–40. 36631537 10.1038/s41391-022-00634-2 PMC10449623

[37] Zambon A, Nguyen TA, Fourcade A, Segalen T, Saout K, Deruelle C, et al. Which protocol for prostate biopsies in patients with a positive MRI? Interest of systematic biopsies by sectors. Prostate Cancer Prostatic Dis. 2024;27:500–6. 10.1038/s41391-023-00770-3 38114598

[38] Lombardo R, Tema G, Nacchia A, Mancini E, Franco S, Zammitti F, et al. Role of perilesional sampling of patients undergoing fusion prostate biopsies. Life. 2023;13:1719. 37629576 10.3390/life13081719 PMC10455324

[39] Delongchamps NB, Portalez D, Brugui ère E, Rouvi ère O, Malavaud B, Mozer P, et al. Are magnetic resonance imaging-transrectal ultrasound guided targeted biopsies noninferior to transrectal ultrasound guided systematic biopsies for the detection of prostate cancer? J Urol. 2016;196:1069–75. 27079582 10.1016/j.juro.2016.04.003

[40] Lee AYM, Chen K, Tan YG, Lee HJ, Shutchaidat V, Fook-Chong S, et al. Reducing the number of systematic biopsy cores in the era of MRI targeted biopsy—implications on clinically-significant prostate cancer detection and relevance to focal therapy planning. Prostate Cancer Prostatic Dis. 2022;25:720–6. 35027690 10.1038/s41391-021-00485-3 PMC9705237

[41] Siddiqui MM, Rais-Bahrami S, Turkbey B, George AK, Rothwax J, Shakir N, et al. Comparison of MR/Ultrasound fusion–guided biopsy with ultrasound-guided biopsy for the diagnosis of prostate cancer. JAMA. 2015;313:390–7. 25626035 10.1001/jama.2014.17942 PMC4572575

[42] Baco E, Ukimura O, Rud E, Vlatkovic L, Svindland A, Aron M, et al. Magnetic resonance imaging–transectal ultrasound image-fusion biopsies accurately characterize the index tumor: correlation with step-sectioned radical prostatectomy specimens in 135 patients. Eur Urol. 2015;67:787–94. 25240973 10.1016/j.eururo.2014.08.077

[43] Bauer JJ, Zeng J, Zhang W, McLeod DG, Sesterhenn IA, Connelly RR, et al. Lateral biopsies added to the traditional sextant prostate biopsy pattern increases the detection rate of prostate cancer. Prostate Cancer Prostatic Dis. 2000;3:43–6. 12497161 10.1038/sj.pcan.4500397

[44] El-Shater Bosaily A, Parker C, Brown LC, Gabe R, Hindley RG, Kaplan R, et al. PROMIS — Prostate MR imaging study: a paired validating cohort study evaluating the role of multi-parametric MRI in men with clinical suspicion of prostate cancer. Contemp Clin Trials. 2015;42:26–40. 25749312 10.1016/j.cct.2015.02.008 PMC4460714

[45] Valerio M, Donaldson I, Emberton M, Ehdaie B, Hadaschik BA, Marks LS, et al. Detection of clinically significant prostate cancer using magnetic resonance imaging–ultrasound fusion targeted biopsy: a systematic review. Eur Urol. 2015;68:8–19. 25454618 10.1016/j.eururo.2014.10.026

[46] Sajadi KP, Kim T, Terris MK, Brown JA, Lewis RW. High yield of saturation prostate biopsy for patients with previous negative biopsies and small prostates. Urology. 2007;70:691–5. 17991539 10.1016/j.urology.2007.05.017

[47] Scattoni V, Zlotta A, Montironi R, Schulman C, Rigatti P, Montorsi F. Extended and saturation prostatic biopsy in the diagnosis and characterisation of prostate cancer: a critical analysis of the literature. Eur Urol. 2007;52:1309–22. 17720304 10.1016/j.eururo.2007.08.006

[48] Irani J, Blanchet P, Salomon L, Coloby P, Hubert J, Malavaud B, et al. Is an extended 20-core prostate biopsy protocol more efficient than the standard 12-core? A randomized multicenter trial. J Urol. 2013;190:77–83. 23313205 10.1016/j.juro.2012.12.109

[49] Holm HH, Gammelgaard J. Ultrasonically guided precise needle placement in the prostate and the seminal vesicles. J Urol. 1981;125:385–7. 7206090 10.1016/s0022-5347(17)55044-2

[50] Sidana A, Blank F, Wang H, Patil N, George AK, Abbas H. Schema and cancer detection rates for transperineal prostate biopsy templates: a review. Ther Adv Urol. 2022;14:17562872221105019. 35783921 10.1177/17562872221105019 PMC9243579

[51] Barzell WE, Melamed MR. Appropriate patient selection in the focal treatment of prostate cancer: the role of transperineal 3-dimensional pathologic mapping of the prostate–a 4-year experience. Urology. 2007;70:27–35. 18194708 10.1016/j.urology.2007.06.1126

[52] Kuru TH, Wadhwa K, Chang RTM, Echeverria LMC, Roethke M, Polson A, et al. Definitions of terms, processes and a minimum dataset for transperineal prostate biopsies: a standardization approach of the Ginsburg Study Group for Enhanced Prostate Diagnostics. BJU Int. 2013;112:568–77. 23773772 10.1111/bju.12132

[53] Ahmed HU, Hu Y, Carter T, Arumainayagam N, Lecornet E, Freeman A, et al. Characterizing clinically significant prostate cancer using template prostate mapping biopsy. J Urol. 2011;186:458–64. 21679984 10.1016/j.juro.2011.03.147

[54] Valerio M, Anele C, Charman SC, van der Meulen J, Freeman A, Jameson C, et al. Transperineal template prostate-mapping biopsies: an evaluation of different protocols in the detection of clinically significant prostate cancer. BJU Int. 2016;118:384–90. 26332050 10.1111/bju.13306

[55] Hansen N, Patruno G, Wadhwa K, Gaziev G, Miano R, Barrett T, et al. Magnetic resonance and ultrasound image fusion supported transperineal prostate biopsy using the ginsburg protocol: technique, learning points, and biopsy results. Eur Urol. 2016;70:332–40. 26995327 10.1016/j.eururo.2016.02.064

[56] Hagens MJ, Fernandez Salamanca M, Padhani AR, van Leeuwen PJ, van der Poel HG, Schoots IG. Diagnostic performance of a magnetic resonance imaging-directed targeted plus regional biopsy approach in prostate cancer diagnosis: a systematic review and meta-analysis. Eur Urol Open Sci. 2022;40:95–103. 35540708 10.1016/j.euros.2022.04.001 PMC9079161

[57] Ahdoot M, Wilbur AR, Reese SE, Lebastchi AH, Mehralivand S, Gomella PT, et al. MRI-targeted, systematic, and combined biopsy for prostate cancer diagnosis. N Engl J Med. 2020;382:917–28. 32130814 10.1056/NEJMoa1910038 PMC7323919

[58] Tschirdewahn S, Wiesenfarth M, Bonekamp D, Püllen L, Reis H, Panic A, et al. Detection of significant prostate cancer using target saturation in transperineal magnetic resonance imaging/transrectal ultrasonography–fusion biopsy. Eur Urol Focus. 2021;7:1300–7. 32660838 10.1016/j.euf.2020.06.020

[59] Saner YM, Wiesenfarth M, Weru V, Ladyzhensky B, Tschirdewahn S, Püllen L, et al. Detection of clinically significant prostate cancer using targeted biopsy with four cores versus target saturation biopsy with nine cores in transperineal prostate fusion biopsy: a prospective randomized trial. Eur Urol Oncol. 2023;6:49–55. 36175281 10.1016/j.euo.2022.08.005

[60] Kim HY, Choi YH, Lee SJ. Effect of sedation anesthesia with intravenous propofol on transrectal ultrasound-guided prostate biopsy outcomes. J Korean Med Sci. 2022;37:e115. 35437964 10.3346/jkms.2022.37.e115 PMC9015899

[61] Altok M, Kim B, Patel BB, Shih YCT, Ward JF, McRae SE, et al. Cost and efficacy comparison of five prostate biopsy modalities: a platform for integrating cost into novel-platform comparative research. Prostate Cancer Prostatic Dis. 2018;21:524–32. 29988098 10.1038/s41391-018-0056-7

[62] Ber Y, Segal N, Tamir S, Benjaminov O, Yakimov M, Sela S, et al. A noninferiority within-person study comparing the accuracy of transperineal to transrectal MRI-US fusion biopsy for prostate-cancer detection. Prostate Cancer Prostatic Dis. 2020;23:449–56. 31953483 10.1038/s41391-020-0205-7 PMC7423592

[63] Loy LM, Lim GH, Leow JJ, Lee CH, Tan TW, Tan CH. A systematic review and meta-analysis of magnetic resonance imaging and ultrasound guided fusion biopsy of prostate for cancer detection-Comparing transrectal with transperineal approaches. Urol Oncol. 2020;38:650–60. 32505458 10.1016/j.urolonc.2020.04.005

[64] Zattoni F, Marra G, Kasivisvanathan V, Grummet J, Nandurkar R, Ploussard G, et al. The detection of prostate cancer with magnetic resonance imaging-targeted prostate biopsies is superior with the transperineal vs. the transrectal approach. A European association of urology-young academic urologists prostate cancer working group multi-institutional study. J Urol. 2022;208:830–7. 36082555 10.1097/JU.0000000000002802

[65] Uleri A, Baboudjian M, Tedde A, Gallioli A, Long-Depaquit T, Palou J, et al. Is there an impact of transperineal versus transrectal magnetic resonance imaging-targeted biopsy in clinically significant prostate cancer detection rate? A systematic review and meta-analysis. Eur Urol Oncol. 2023;6:621–8. 37634971 10.1016/j.euo.2023.08.001

[66] Wu Q, Tu X, Zhang C, Ye J, Lin T, Liu Z, et al. Transperineal magnetic resonance imaging targeted biopsy versus transrectal route in the detection of prostate cancer: a systematic review and meta-analysis. Prostate Cancer Prostatic Dis. 2023;2:1–10. 10.1038/s41391-023-00729-4 37783837

[67] Bennett HY, Roberts MJ, Doi SAR, Gardiner RA. The global burden of major infectious complications following prostate biopsy. Epidemiol Infect. 2016;144:1784–91. 26645476 10.1017/S0950268815002885 PMC9150640

[68] Berry B, Parry MG, Sujenthiran A, Nossiter J, Cowling TE, Aggarwal A, et al. Comparison of complications after transrectal and transperineal prostate biopsy: a national population-based study. BJU Int. 2020;126:97–103. 32124525 10.1111/bju.15039

[69] Mian BM, Feustel PJ, Aziz A, Kaufman RP, Bernstein A, Fisher HAG. Clinically significant prostate cancer detection following transrectal and transperineal biopsy: results of the prostate biopsy efficacy and complications randomized clinical trial. J Urol. 2024;212:21–31. 38700844 10.1097/JU.0000000000003979

[70] Hu JC, Assel M, Allaf ME, Ehdaie B, Vickers AJ, Cohen AJ, et al. Transperineal versus transrectal magnetic resonance imaging-targeted and systematic prostate biopsy to prevent infectious complications: the PREVENT randomized trial. Eur Urol. 2024;S0302-2838(23)03342-0. 10.1016/j.eururo.2023.12.015 PMC11976521 38212178

[71] Castellani D, Pirola GM, Law YXT, Gubbiotti M, Giulioni C, Scarcella S, et al. Infection rate after transperineal prostate biopsy with and without prophylactic antibiotics: results from a systematic review and meta-analysis of comparative studies. J Urol. 2022;207:25–34. 34555932 10.1097/JU.0000000000002251

[72] Wegelin O, Exterkate L, van der Leest M, Kelder JC, Bosch JLHR, Barentsz JO, et al. Complications and adverse events of three magnetic resonance imaging–based target biopsy techniques in the diagnosis of prostate cancer among men with prior negative biopsies: results from the FUTURE Trial, a multicentre randomised controlled trial. Eur Urol Oncol. 2019;2:617–24. 31519516 10.1016/j.euo.2019.08.007

[73] Nassiri N, Chang E, Lieu P, Priester AM, Margolis DJA, Huang J, et al. Focal therapy eligibility determined by magnetic resonance imaging/ultrasound fusion biopsy. J Urol. 2018;199:453–8. 28830754 10.1016/j.juro.2017.08.085 PMC5780241

[74] Johnson DC, Yang JJ, Kwan L, Barsa DE, Mirak SA, Pooli A, et al. Do contemporary imaging and biopsy techniques reliably identify unilateral prostate cancer? Implications for hemiablation patient selection. Cancer. 2019;125:2955–64. 31042322 10.1002/cncr.32170 PMC7368458

[75] Choi YH, Lee CU, Song W, Chang Jeong B, Seo SI, Jeon SS, et al. Combination of multiparametric magnetic resonance imaging and transperineal template-guided mapping prostate biopsy to determine potential candidates for focal therapy. Prostate Int. 2023;11:100–6. 37409092 10.1016/j.prnil.2022.12.003 PMC10318325

[76] Ong S, Chen K, Grummet J, Yaxley J, Scheltema MJ, Stricker P, et al. Guidelines of guidelines: focal therapy for prostate cancer, is it time for consensus? BJU Int. 2023;131:20–31. 36083229 10.1111/bju.15883 PMC10087270

[77] Levine MA, Ittman M, Melamed J, Lepor H. Two consecutive sets of transrectal ultrasound guided sextant biopsies of the prostate for the detection of prostate cancer. J Urol. 1998;159:471–5. 9649265 10.1016/s0022-5347(01)63951-x

[78] Stewart CS, Leibovich BC, Weaver AL, Lieber MM. Prostate cancer diagnosis using a saturation needle biopsy technique after previous negative sextant biopsies. J Urol. 2001;166:86–91. 11435830

[79] Kaplan I, Oldenburg NE, Meskell P, Blake M, Church P, Holupka EJ. Real time MRI-ultrasound image guided stereotactic prostate biopsy. Magn Reson Imaging. 2002;20:295–9. 12117612 10.1016/s0730-725x(02)00490-3

[80] Bourne R, Katelaris P, Danieletto S, Dzendrowskyj T, Stanwell P, Mountford C. Detection of prostate cancer by magnetic resonance imaging and spectroscopy in vivo. ANZ J Surg. 2003;73:666–8. 12887547 10.1046/j.1445-2197.2003.02700.x

[81] Beyersdorff D, Winkel A, Hamm B, Lenk S, Loening SA, Taupitz M. MR imaging-guided prostate biopsy with a closed MR unit at 1.5 T: initial results. Radiology. 2005;234:576–81. 15616117 10.1148/radiol.2342031887

[82] Barrett T, Patterson AJ, Koo BC, Wadhwa K, Warren AY, Doble A, et al. Targeted transperineal biopsy of the prostate has limited additional benefit over background cores for larger MRI-identified tumors. World J Urol. 2016;34:501–8. 26238348 10.1007/s00345-015-1650-0 PMC4799791

[83] Emiliozzi P, Longhi S, Scarpone P, Pansadoro A, DePaula F, Pansadoro V. The value of a single biopsy with 12 transperineal cores for detecting prostate cancer in patients with elevated prostate specific antigen. J Urol. 2001;166:845–50. 11490231

